# EXPLORATION OF THE NEED FOR GRIEF SERVICES: METHODOLOGY OF A COMMUNITY-BASED RESEARCH STUDY

**DOI:** 10.1093/geroni/igad104.1783

**Published:** 2023-12-21

**Authors:** Lisa Stone-Bury

**Affiliations:** University of Colorado Colorado Springs, Colorado Springs, Colorado, United States

## Abstract

Within the Exploration, Preparation, Implementation, and Sustainment (EPIS) framework, community-based research (CBR) is a key component of Exploration and Preparation. This presentation describes the process of conducting a CBR study on grief service needs within Colorado Springs. A community-academic partnership was formed, resulting in a multidisciplinary team of clinical geropsychology academics, a chaplain, a mental health professional, and a funeral director, with the goal of launching a non-profit grief center that catered to identified local needs. Challenges and successes in this partnership will be explored. Two surveys were created: (1) community-wide survey targeting adults across the lifespan and (2) professional survey targeting individuals who interface with grief in their profession (e.g., healthcare providers, educators, lawyers, faith leaders). The authors facilitated survey development that balanced practical needs with existing empirical literature on grief services. Qualitative and quantitative measurement focused both on past experiences with grief (to understand the community’s history) and use of future grief services (to understand the types of services most desired by the community). The multidisciplinary team pooled resources related to recruitment and dissemination of the surveys, combining traditional research recruitment practices (e.g., undergraduates, research database of older adults) with networks of local community leaders (e.g., places of worship, local healthcare agencies, newspapers). These CBR efforts resulted in large samples of community members (n = 668; age range = 18 to 65+) and professionals (n = 141). Ultimately, this CBR study laid important groundwork to prepare the multidisciplinary team to match their services to the community’s expressed needs.

